# Fc Gamma Receptor IIIB (FcγRIIIB) Polymorphisms Are Associated with Clinical Malaria in Ghanaian Children

**DOI:** 10.1371/journal.pone.0046197

**Published:** 2012-09-25

**Authors:** Bright Adu, Daniel Dodoo, Selorme Adukpo, Paula L. Hedley, Fareed K. N. Arthur, Thomas A. Gerds, Severin O. Larsen, Michael Christiansen, Michael Theisen

**Affiliations:** 1 Department of Clinical Biochemistry and Immunology, Statens Serum Institut, Copenhagen, Denmark; 2 Centre for Medical Parasitology at Department of International Health, Immunology, and Microbiology and Department of Infectious Diseases, Rigshospitalet, University of Copenhagen, Copenhagen, Denmark; 3 Noguchi Memorial Institute for Medical Research, University of Ghana, Legon, Ghana; 4 Department of Biomedical Sciences, University of Stellenbosch, Cape Town, South Africa; 5 Department of Biochemistry and Biotechnology, Kwame Nkrumah University of Science and Technology, Kumasi, Ghana; 6 Department of Biostatistics, University of Copenhagen, Copenhagen, Denmark; Institut national de la santé et de la recherche médicale - Institut Cochin, France

## Abstract

*Plasmodium falciparum* malaria kills nearly a million people annually. Over 90% of these deaths occur in children under five years of age in sub-Saharan Africa. A neutrophil mediated mechanism, the antibody dependent respiratory burst (ADRB), was recently shown to correlate with protection from clinical malaria. Human neutrophils constitutively express Fc gamma receptor-FcγRIIA and FcγRIIIB by which they interact with immunoglobulin (Ig) G (IgG)-subclass antibodies. Polymorphisms in exon 4 of *FCGR2A* and exon 3 of *FCGR3B* genes encoding FcγRIIA and FcγRIIIB respectively have been described to alter the affinities of both receptors for IgG. Here, associations between specific polymorphisms, encoding FcγRIIA p.H166R and FcγRIIIB-NA1/NA2/SH variants with clinical malaria were investigated in a longitudinal malaria cohort study. FcγRIIA-p.166H/R was genotyped by gene specific polymerase chain reaction followed by allele specific restriction enzyme digestion. *FCGR3B*-exon 3 was sequenced in 585 children, aged 1 to 12 years living in a malaria endemic region of Ghana. Multivariate logistic regression analysis found no association between FcγRIIA-166H/R polymorphism and clinical malaria. The A-allele of *FCGR3B*-c.233C>A (rs5030738) was significantly associated with protection from clinical malaria under two out of three genetic models (additive: *p* = 0.0061; recessive: *p* = 0.097; dominant: *p* = 0.0076) of inheritance. The FcγRIIIB-SH allotype (CTG**A**AA) containing the 233A-allele (in bold) was associated with protection from malaria (*p* = 0.049). The FcγRIIIB-NA2*03 allotype (CTGCGA), a variant of the classical FcγRIIIB-NA2 (CTGCAA) was associated with susceptibility to clinical malaria (*p* = 0.0092). The present study is the first to report an association between a variant of FcγRIIIB-NA2 and susceptibility to clinical malaria and provides justification for further functional characterization of variants of the classical FcγRIIIB allotypes. This would be crucial to the improvement of neutrophil mediated functional assays such as the ADRB assay aimed at assessing the functionality of antibodies induced by candidate malaria vaccines.

## Introduction

*Plasmodium falciparum* malaria kills nearly a million people annually and over 90% of deaths occur in children under five years of age in sub-Saharan Africa [1]. Several sero-epidemiological studies have associated *P. falciparum* antigen-specific cytophilic immunoglobulin (Ig) G (particularly IgG1 and IgG3) with protection from clinical malaria [2]–[4] suggesting a critical role for immune effector cells in malaria immunity. Monocytes, upon activation by IgG opsonized infected erythrocytes are thought to release certain, as yet uncharacterized, factors that inhibit intra-erythrocytic parasite growth [5], [6]. This mechanism, termed antibody dependent cellular inhibition (ADCI), has been widely studied in *in vitro* systems but has so far not shown any significant correlation with protection against clinical malaria. On the other hand a correlation has been reported between neutrophil mediated antibody dependent respiratory burst (ADRB) and protection from clinical malaria in two Senegalese populations which differ in malaria transmission intensity [7]. Human neutrophils constitutively express two receptors, namely Fc gamma receptor (FcγR)- FcγRIIA and FcγRIIIB [8], which bind the Fc domain of IgG. These receptors complement each other functionally [9]. FcγRIIA is a transmembrane protein while FcγRIIIB has no transmembrane domain and is anchored in the plasma membrane through a C-terminus linked glycosylphosphatidylinositol moiety [10], [11]. In addition to neutrophils, most cells of the immune system, including monocytes, macrophages, eosinophils, basophils, Langerhans cells, platelets, placental endothelial cells and some T cells subpopulations are known to express FcγRIIA while FcγRIIIB is expressed exclusively on neutrophils [12]. Crosslinking of FcγRIIA on neutrophils induces phagocytosis of IgG-opsonised particles [13] while FcγRIIIB crosslinking leads to neutrophil degranulation and generation of reactive oxygen species (ROS), which have been shown to alter FcγRIIA avidity and efficiency in an allele-specific manner [14]. ROS are known to be highly toxic to intra-erythrocytic malaria parasites [5], [15], [16] and high ROS production by neutrophils has been correlated with fast *P. falciparum* clearance in Gabonese children [17] and protection from clinical malaria in two Senegalese populations [7].

Single nucleotide polymorphisms (SNPs) which alter the affinities of both FcγRIIA and FcγRIIIB in binding IgG subclasses have been described. In the present study, we hypothesised that the implications of these polymorphisms in the described neutrophil mediated immune correlate of clinical protection from malaria (the ADRB mechanism) by Joos and colleagues [7], could be critical to the outcome of *P. falciparum* infection. In the *FCGR2A* gene (NM_021642.3), a non-synonymous variant, c.497G>A (rs1801274) in exon 4, which specifies the ligand binding domain of the receptor, causes an arginine (R) substitution of an histidine (H) amino acid at position 166 of the polypeptide (p.H166R) [18]. The FcγRIIA-H166 allele has higher affinity for human IgG2 and IgG3, compared to FcγRIIA-R166 which binds weakly [19]. In a recent study, Schuldt and colleagues found FcγRIIA-R166/R166 homozygosity to be associated with severe malarial anaemia in Ghanaian children [20]. FcγRIIIB bears the neutrophil antigen (NA) polymorphism in its membrane-distal Ig-like domain and is found in three polymorphic forms, called human neutrophil antigen (HNA)-1a (or NA1), HNA-1b (or NA2) and HNA-1c (or SH), which are encoded by FCGR3B*1, FCGR3B*2 and FCGR3B*3 alleles, respectively [21]. The FCGR3B*1 and FCGR3B*2 alleles differ in five nucleotide positions; c.108C>G; c.114T>C; c.194A>G; c.244A>G and c.316A>G [22] in exon 3 of the *FCGR3B* gene (NM_00570.4) which results in four amino acid changes at positions; p.S36R; p.N65S; p.N82D and p.I106V of the peptide chain with the c.114T>C being a synonymous coding SNP [23]. FcγRIIIB-NA1 facilitates phagocytosis of IgG1- and IgG3-opsonized particles more efficiently than FcγRIIIB-NA2. This may be due to the presence of two additional N-linked glycosylation sites in FcγRIIIB-NA2 compared to FcγRIIIB-NA1 [24]. The nucleotide sequence of the FcγRIIIB coding region of FCGR3B*3 is identical to the FCGR3B*2 sequence except for a SNP c.233C>A (rs5030738) encoding p.A78D resulting in the expression of the FcγRIIIB-SH allo-antigen [25]. This allotype has been associated with about a third of FcγRIIIB alleles and higher expression levels of FcγRIIIB but its influence on receptor function is unclear [26]. Furthermore, in individuals whose neutrophils lack the FcγRIIIB molecule (NA_null_) the corresponding gene deletion has been described [27]. Sequencing analysis has identified variants of the classical FcγRIIIB-NA1/NA2 allotypes but their functional significance have, as yet, not been characterised [28]–[30]. The FcγRIIIB-NA2 allotype in combination with the FcγRIIA-166H allele have been associated with cerebral malaria in Thai individuals [31] and severe malarial anaemia in Kenyan children [32]. In general, malaria immunogenetic studies have so far mainly focused on severe forms of malaria using either cross-sectional and/or case-control study data [20], [32]–[34]. In the present study, we successfully elucidated associations between *FCGR3B* and *FCGR2A* polymorphisms and clinical malaria using data from a well characterised longitudinal cohort study.

## Results

### Demographic and clinical characteristics of study population

Of the 669 out of 798 children who successfully completed the 42 week longitudinal follow up, DNA was available for 585 (87.4%). These children were distributed across six villages as follows: Asutsuare (ASU) (169), Kewum (KEW) (138), Avakpo (AVA) (33), Mafikorpe (MAF) (36), Osuwem (OSU) (71) and Volivo (VOL) (138) (Table 1). A total of 329 (56.2%) children were ≤5 years of age and 88 (15.0%) were sickle cell positive. The study population consisted predominantly of the Ga-Adangbe (n = 430, 73.5%) and Ewe (n = 76, 13.0%) ethnic groups. The remaining (n = 79, 13.5%), children belonged to the Akan, Hausa or Fulani ethnic groups. Children who used bed net constituted 42.1% of the study population. The gender distribution was not significantly different between the villages (*p* = 0.75, χ^2^ analysis) while the distribution of age, blood group, ethnicity and sickle cell status were significantly different between the villages (*p*≤0.0007, χ^2^ analysis) (Table 1).

**Table 1:** Demographics and clinical characteristics of study participants.

	ASU	KEW	AVA	MAF	OSU	VOL	*p*-value¥
**N**	169	138	33	36	71	138	
**Age group (years)**							
1–5	122	66	17	15	50	59	
6–12	47	72	16	21	21	79	<0.0001
**Sex**							
Male	83	71	19	17	32	62	
Female	86	67	14	19	39	76	0.75
**Sickle cell**							
Negative	152	129	12	23	68	113	
Positive	17	9	21	13	3	25	<0.0001
**Blood group**							
O	96	64	10	11	37	62	
A	33	31	14	7	8	18	
B	32	36	9	13	19	46	
AB	8	7	0	5	7	12	0.0007
**Bed net use**							
Yes	63	65	21	26	26	45	
No	106	73	12	10	45	93	<0.0001
**Ethnic group**							
Ga-Adangbe	124	103	15	14	67	107	
Ewe	15	7	14	18	1	21	
Other	30	28	4	4	3	10	<0.0001
**Clinical malaria status**							
Susceptible	12	15	2	0	9	14	
Protected	157	123	31	36	62	124	0.24

¥ *p*-values refer to chi-square tests. ASU: Asutsuare; KEW: Kewum-Atrobinya; AVA: Avakpo; MAF: Mafikorpe; OSU: Osuwem; VOL: Volivo.

### P. falciparum infections in the study cohort

The incidence of clinical malaria during the follow up period was low (52 cases, 8.9%). These individuals were considered susceptible to clinical malaria while those who never had clinical malaria were considered protected. The protected group was sub-categorized into two: (1) any individual with no malaria episode and (2) only individuals with no malaria episodes but with parasites detectable by microscopy at any time point during the follow up period. The overall number of susceptible and protected individuals did not differ significantly (*p* = 0.24, χ^2^ analysis) among the six villages (Table 1). Logistic regression analyses investigating the association of the covariates, age groups, sex, blood group, ethnicity and sickle cell status with clinical malaria, found an association with ethnicity. Individuals of the Ewe ethnic group had a significantly reduced risk of clinical malaria compared to other ethnic groups (likelihood ratio test: *p*_(LR test)_ = 0.013) (Table 2).

**Table 2:** Covariates association with clinical malaria.

Covariates	Susceptible (n = 52)	Protected (n = 533)	OR(95%CI)^a^	*p*-value^a^	LR test *p*-value
**Age group (years)**					
1–5	29	300	1		
6–12	23	233	1.01 (0.56–1.81)	0.97	0.97
**Sex**					
Male	25	259	1		
Female	27	274	0.96 (0.54–1.72)	0.89	0.89
**Sickle cell**					
Negative	45	452	1		
Positive	7	81	0.94 (0.37–2.07)	0.89	0.89
**Blood group**					
O	26	254	1		
A	6	105	0.59 (0.22–1.42)	0.27	
B	15	140	1.08 (0.54–2.11)	0.81	
AB	5	34	1.58 (0.50–4.16)	0.39	0.46
**Bed net use**					
Yes	20	226	1		
No	32	307	1.06 (0.58–1.96)	0.85	0.85
**Ethnic group**					
Other	8	71	1		
Ga-Adangbe	43	387	0.97 (0.46–2.33)	0.95	
Ewe	1	75	0.12 (0.01–0.67)	0.047	0.013

Odds Ratios (OR) and 95% confidence intervals (CI) were determined using multivariate logistic regression.

a Analysis for each covariate was adjusted by the other remaining covariates: age groups, sex, sickle cell status, blood group, bed net use and ethnic group. The likelihood ratio (LR) test result compares the adjusted model to a model which only includes the adjusting factors and thereby tests if the variable has an effect on susceptibility.

### Distribution of FCGR2A and FCGR 3B genotype in Ghanaian children

The FcγRIIA-p.H166R (c.497A>G) genotypes were determined by gene specific polymerase chain reaction amplification followed by allele specific restriction enzyme digestion (PCR-ASRED). The minor allele frequency (which is this population was the A-allele) was 41.0% and there was no deviation from HWE (*p* = 0.690, χ^2^ analysis). Nucleotide sequencing of exon 3 of *FCGR3B* identified the six polymorphisms (c.108C>G, c.114T>C, c.194A>G, c.233C>A, c.244A>G and c.316A>G), defining the FcγRIIIB-NA1/NA2/SH allotypes. The previously described c.197T>G, and c.297G>T polymorphisms were also found in 87 (14.9%) and 46 (7.9%) individuals respectively. A new SNP, c.232G>A was identified in five (0.85%) of the study participants. All the SNPs were in HWE, *p*>0.05 (Table 3). The distribution of the genotypes was not significantly different between the ethnic groups except for the *FCGR2A*-c.497A>G (*p* = 0.038, χ^2^ test) and *FCGR3B*-c.194A>G (*p* = 0.017, χ^2^ test) SNPs (Table S1).

**Table 3:** *FCGR3B* allele frequencies and Hardy-Weinberg (HW) estimations in protected individuals.

Variation ID	Alleles*	Amino acid change*	Minor allele (Frequency)	HW *p*-value^a^
**rs403016**	c.108C>G	p.S36R	G (0.491)	1.00
**rs447536**	c.114T>C	Synonymous coding	C (0.471)	0.62
**rs448740**	c.194A>G	p.N65S	A (0.390)	0.61
**rs5030738**	c.233C>A	p.A78D	A (0.214)	0.49
**rs428888**	c.244A>G	p.N82D	A (0.491)	0.066
**rs2290834**	c.316A>G	p.I106V	G (0.251)	0.29

* Allele and amino acid numberings refer to positions in *FCGR3B* transcript ENST00000367964.

a HW estimations based on children (n = 267) who had diploid copies of *FCGR3B*, were the first sibling in a family and were not susceptible to clinical malaria in the observation period.

### FCGR2A and FCGR 3B genotypes and clinical malaria

The distribution of the *FCGR2A*-c.497A>G (FcγRIIA-p.H166R) genotype frequencies among the susceptible and protected groups did not differ significantly in a multivariate analysis and had no influence on the outcome of *P. falciparum* infection (*p*_(LR test)_ = 0.80) (Table S2). The *FCGR3B*-c.233C>A (FcγRIIIB-p.A78D) polymorphism showed a statistically significant association with the outcome of *P. falciparum* infection (*p*_(LR test)_ = 0.009) (Table S1). The A-allele was significantly associated with protection from clinical malaria under two out of the three genetic models of inheritance (additive: *p* = 0.0061, recessive: *p* = 0.097 and dominant: *p* = 0.0076) analysed using a control group that included all individuals with no clinical malaria during follow up (Table 4). The confounding effect of possible heterogeneity in exposure was investigated by repeating the analysis with a redefined control group comprising only individuals with no malaria episodes but with a definitive evidence of exposure ie. with parasites detected by microscopy during follow up. The same marker (c.233A-allele) was significantly associated with protection from malaria (Table S3) under the same genetic models of inheritance previously observed, confirming the initial observation. Disease association analyses for *FCGR3B* were restricted to the six SNPs encoding the FcγRIIIB-NA1/NA2/SH allotypes. Of the three additional SNPs, only the T-allele of c.297G>T was significantly associated with protection from clinical malaria (OR = 0.307, 95%CI = 0.16–0.61, *p* = 0.0016, *Fisher's* exact test).

**Table 4:** Single marker association of *FCGR3B* alleles with clinical malaria.

		MAF		Additive model		Recessive model		Dominant model	
SNP ID	Minor Allele	ProtectedŦŦ	Susceptible	OR (95% CI)	*p*-value	OR (95% CI)	*p*-value	OR (95% CI)	*p*-value
rs403016	C	0.49	0.50	1.04 (0.71–1.54)	0.84	1.38 (0.74–2.53)	0.31	0.82 (0.44–1.53)	0.53
rs447536	C	0.47	0.46	0.98 (0.67–1.45)	0.94	1.11 (0.57–2.13)	0.77	0.88 (0.47–1.63)	0.68
rs448740	A	0.40	0.38	0.95 (0.64–1.42)	0.82	1.16 (0.56–2.40)	0.69	0.80 (0.45–1.44)	0.46
rs5030738	A	0.22	0.10	0.43 (0.23–0.78)	0.0061	0.18 (0.02–1.36)	0.097	0.37 (0.18–0.77)	0.0076
rs428888	A	0.50	0.40	0.74 (0.51–1.06)	0.10	0.52 (0.25–1.07)	0.075	0.74 (0.40–1.36)	0.34
rs2290834	G	0.27	0.22	0.81 (0.52–1.27)	0.36	0.87 (0.34–2.20)	0.77	0.69 (0.37–1.27)	0.23

Odds ratio (OR) and 95% confidence intervals (CI) were determined using multivariate logistic regression controlling for age, gender, ethnicity, sickle-cell status, *FCGR3B* copy number, blood group, family structure and use of bed net. MAF: minor allele frequency.

Ŧ All individuals who never had malaria despite parasitaemia at any time point (monthly blood slide) during the study, plus all individuals who never had malaria but without detectable parasitaemia by microscopy.

### FCGR3B haplotype association with clinical malaria

**Figure 1:**
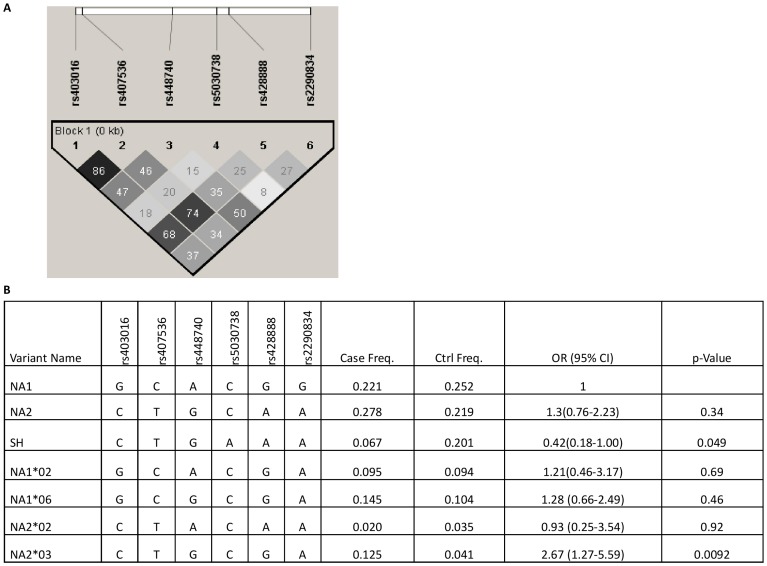
Studied SNPs in the *FCGR3B* gene, linkage disequilibrium (LD) patterns and haplotype association analysis. **A**) A schematic of exon 3 *FCGR3B* gene (**NM_000570.4**) and LD plot of the respective SNPs visualised using Haploview v4.2. The LD plot shows pairwise r^2^ values (×100) given in the squares for each comparison between the SNPs. White squares represent r^2^ values equal to 0. Different shades of grey represent r^2^ values between 0 and 1. **B**) Haplotype associations with susceptibility to clinical malaria compared to clinically protected individuals. Odds ratio (OR) and 95% confidence intervals (CI) were determined using multivariate logistic regression controlling for age, gender, ethnicity, sickle-cell status, *FCGR3B* copy number, blood group and use of bed net. The haplotype with the highest frequency in the study population was considered the reference group in the multivariate logistic regression analyses. ^Ŧ^Variant first reported in this study, the associated gene for NA1*06 is FCGR3B*01A194G, G316A; ^Ψ^ Variants first reported by Matsuo et al, [28], the associated genes for NA1*02, NA2*02 and NA2*03 are FCGR3B*01G316A, FCGR3B*02G194A and FCGR3B*02A244G respectively.

The six SNPs defining the FcγRIIIB-NA1/NA2/SH were investigated for association with clinical malaria. Pairwise r^2^ values between these SNPs (0.08≤r^2^≤0.86) from linkage analysis revealed a significant linkage between most of the SNPs (Figure 1A). A haplotype block was defined to include all SNPs in order to estimate the frequencies of FcγRIIIB allotypes in the entire study population (Figure 1A). The haplotype analysis identified the three major FcγRIIIB allotypes (FcγRIIIB-NA1/NA2/SH), two variants of FcγRIIIB-NA1 (FcγRIIIB-NA1*02 and FcγRIIIB-NA1*06) and two variants of FcγRIIIB-NA2 (FcγRIIIB-NA2*02 and FcγRIIIB-NA2*03) (Figure 1B). The FcγRIIIB-NA2*03 was significantly associated with susceptibility to clinical malaria (OR = 2.67, 95%CI = 1.27–5.59, *p* = 0.0092) while the FcγRIIIB-SH allotype showed a borderline significant association with protection (OR = 0.42, 95%CI = 0.18–1.00, *p* = 0.049) (Figure 1B). When only individuals who did not have malaria but with detectable parasites during the follow up period were considered as controls in the haplotype association analysis, logistic regression could not be performed because the risk haplotype FcγRIIIB-NA2*03 was completely absent in the control group (n = 57).

### Population differences of FCGR3B-c.233C>A polymorphism

**Figure 2:**
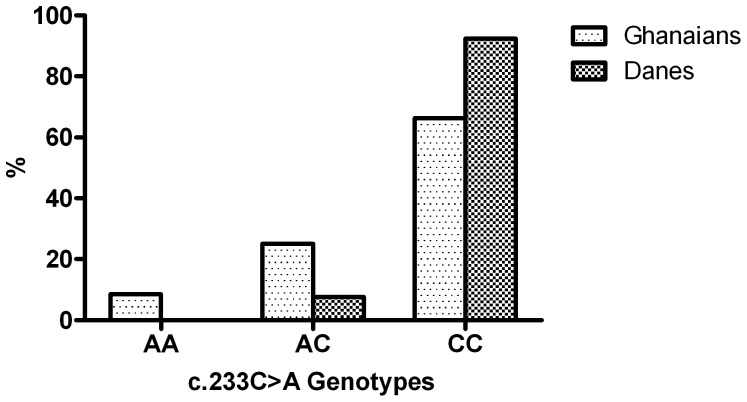
*FCGR3B*-c.233C>A genotypes. Genotype distribution compared between malaria endemic (Ghanaian) and malaria non endemic (Danish) populations.

Exon 3 of *FCGR3B* was sequenced in 132 native Danish blood donors and genotype distributions compared to the Ghanaian population to investigate differences and possible selection. Sequence analysis found all the six SNPs defining the FcγRIIIB-NA1/NA2/SH system. All SNPs were in HWE (*p*>0.05). One person carried synonymous variant (c.201C>T). The c.233C>A genotype frequencies were significantly different (*p*<0.0001, χ^2^ analysis) between the Ghanaian and Danish populations (Figure 2). Although the A-allele remained the minor allele in both populations, none of the 132 Danes was homozygous compared to the 8.5% homozygosity in the Ghanaian population. Only 10 individuals were heterozygous in the Danish population. The Tajima's D statistic estimated for *FCGR3B* exon 3 showed a significant positive deviation from the values expected under neutrality in both the Ghanaian (D = 2.9, *p*<0.05) and Danish (D = 2.2, *p*<0.05) populations. Comparisons with data from the 1000 Genomes Project database showed that the A-allele frequency for the African (AFR) (17.9%) super population was similar to that in the Ghanaians (21.4%) in this study. Similarly, the A-allele frequency for the Ad Mixed American (AMR) (3.3%) and European (EUR) (0.8%) super populations were comparable to the Danes (3.8) (Table 5). Thus, the A-allele, which was associated with protection from *P. falciparum* malaria, was more frequent in malaria endemic populations than in non-endemic populations. To further evaluate the extent of divergence with respect to this polymorphism, pairwise F_ST_ indices were calculated for all five populations (Ghanaians, Danes, AFR, AMR and EUR). The AFR and the Ghanaian population shared a pairwise F_ST_ value of 0.004 while all comparisons between the malaria endemic (Ghanaians and AFR) and the malaria non-endemic (Danes, AMR and EUR) populations yielded consistently higher F_ST_ values (0.098≤F_ST_≤0.194) (Table 6).

**Table 5:** rs5030738 (c.233C>A) allele distribution among malaria endemic and malaria non-endemic populations.

	Malaria endemic		Malaria non-endemic		
rs5030738 c.233C>A	Ghanaians (n = 585)	AFR* (n = 248)	Danes (n = 132)	EUR* (n = 381)	AMR* (n = 181)
**A-allele %**	21.4	17.9	3.8	0.8	3.3
**C-allele %**	78.6	82.1	96.2	99.2	96.7

* Allele frequency data from the 1000 Genomes project data base. AFR: African; AMR: Ad Mixed American; EUR: European.

**Table 6:** Pairwise genetic distances between malaria versus non-malaria population with respect to c.233C>A (rs5030738) polymorphism.

Population	AFRŦ	Danes	EURŦ	AMRŦ
**Ghanaian**	0.004	0.131	0.194	0.141
**AFR** Ŧ		0.098	0.159	0.106
**Danes**			0.020	0.000
**EUR** Ŧ				0.015

F_ST_ distance (Latter et al., 1972).

Ŧ Allele frequency data from the 1000 Genomes Project database (http://browser.1000genomes.org/Homo_sapiens/Variation/Population?db=coreg=ENSG00000162747r=1:161592986-161601753t=ENST00000531221v=rs5030738vdb=variationvf=8673417). AFR: African; AMR: Ad Mixed American; EUR: European.

## Discussion

Using PCR-ASRED and exon 3 nucleotide sequencing data for *FCGR2A* and *FCGR3B* respectively, the association between SNPs which alter the affinity of these receptors for IgG subclasses and clinical malaria were studied in a cohort of Ghanaian children. There was no association between the *FCGR2A*-c.497A>G (FcγRIIA-p.H166R) polymorphism and clinical malaria while the A-allele of *FCGR3B*-c.233C>A polymorphism (rs5030738) was strongly associated with protection from clinical malaria. Haplotype analysis identified the FcγRIIIB-SH allotype (CTG**A**AA) containing the c.233A-allele (in bold) to be associated with protection while a variant of FcγRIIIB-NA2, the FcγRIIIB-NA2*03 allotype (CTGCGA) was associated with susceptibility to clinical malaria. Individuals of the Ewe ethnic group showed a reduced risk to clinical malaria compared to other ethnic groups, however, the distribution of genotypes was not different between the ethnic groups except for *FCGR2A*-497A>G and *FCGR3B*-194A>G. These genotypes had no association with clinical malaria. Also, while the ethnic group distribution was different between the villages, the number of susceptible and protected individuals was not different between the villages. Thus, the genetic associations with protection and susceptibility observed here could not be due to a bias caused by ethnicity. Heterogeneity in malaria exposure is an important confounder in association studies [35]. In the current study, the outcome of the association analysis did not differ whether or not the control group included individuals with no malaria and no detectable asymptomatic parasitaemia by microscopy. This suggests exposure may have been homogeneous and that asymptomatic malaria infection in some of the individuals went undetected due to immunity resulting in sub-microscopic parasitaemia [35]. The protective allele (c.233A), though a minor allele in all populations in this study, still had a significantly higher frequency in malaria endemic populations compared to the non-endemic populations, possibly because of positive selection by *P. falciparum* malaria. The Tajima's D estimates show that *FCGR3B* exon 3 is under strong selection pressure in the Ghanaian populations and also to a lesser extent in the Danish population.

Both sero-epidemiological [2]–[4] and *in vitro* studies [5], [36] have demonstrated that cytophilic IgG (1 and 3) subclasses are the most important in controlling parasites multiplication and/or disease, emphasizing the importance of effector cells such as monocytes and neutrophils in malaria immunity. Neutrophils are the most abundant leucocytes and known to have both high phagocytic and cytotoxic capabilities through the generation and release of potent cytotoxic mediators such as reactive oxygen species (ROS) and proteases [37]. They are the main effector cells in the ADRB mechanism recently shown to correlate with protection from clinical malaria [7]. Neutrophils engage IgG1 and IgG3 immune complexes (ICs) through FcγRIIA and FcγRIIIB constitutively expressed on the cell surface. Although these receptors function synergistically, it has been shown that phagocytosis by neutrophils is primarily dependent on FcγRIIA crosslinking [13] while ROS release is by FcγRIIIB crosslinking with ICs [14]. The lack of association between clinical malaria and any of the genotypes of FcγRIIA-p.H166R in this study suggests that, neutrophil ROS activity may be paramount to phagocytic activity in protecting against clinical malaria since only FcγRIIIB polymorphisms could explain the outcome of *P. falciparum* infection. This notion is supported by both *in vitro* studies of ROS toxicity on intra-erythrocytic malaria parasites [5], [15], [16] and field studies where high ROS production by neutrophils correlated with fast *P. falciparum* clearance [17] and protection from clinical malaria [7].

The c.233A-allele of *FCGR3B* which was associated with protection from clinical malaria in the present study causes a replacement of the hydrophobic amino acid alanine (A) with the negatively charged aspartic acid (D) at position 78 in the NA2 protein. This substitution results in the SH allotype which also showed association with protection. The functional implications of this p.A78D substitution in receptor-ligand (antibody) interactions have not been conclusively shown. Koene and colleagues [26] suggest it may influence a ligand epitope possibly located in the membrane distal Ig-like domain. In the same study, the SH allotype was associated with high expression levels of FcγRIIIB. Thus, protection from clinical malaria seen in individuals with the A-allele and SH allotype may be due to increased ROS production as a result of both enhanced antibody-receptor interaction and increased FcγRIIIB density on neutrophil cell surface. The maximum statistical power for A-allele association with protection was observed under the dominant model with homozygous individuals having over 60% reduced risk to acquiring clinical malaria compared to the other models tested. In genetic association studies, maximum power to detect significant association is reached when the ‘true’ mode of inheritance and the genetic model used in the analysis are concordant [38]. Thus, the dominant model best explains the mode of inheritance of the c.233C>A polymorphism in the Ghanaian cohort studied.

Association studies based on Polymerase Chain Reaction-Sequence Specific Primer (PCR-SSP) data of *FCGR2A* and *FCGR3B* have found individuals carrying the NA2 allotype in combination with FcγRIIA-166H have an increased risk of developing cerebral malaria [31] and severe malarial anaemia [32]. Here, using *FCGR3B*-exon 3 nucleotide sequencing data, we find a previously reported variant of FcγRIIIB-NA2, FcγRIIIB-NA2*03 (associated gene: FCGR3B*02A244G, [28]) to be associated with susceptibility to clinical malaria. While the PCR-SSP technique [39] is an established method for genotyping *FCGR3B* and has been extensively used, sequencing data [28]–[30], [40] have consistently shown that the *FCGR3B* gene is more polymorphic than was previously thought. Variations at the allotype defining sites could result in aberrant PCR-SSP typing [30], [40]. More importantly, the PCR-SSP method cannot differentiate between the classical NA2 and the variant NA2*03 and will simply type both as NA2. Thus, the PCR-SSP method alone may not be enough to sufficiently characterise FcγRIIIB allotypes in disease association studies. It is not clear how exon 3 sequencing data would have affected the conclusions from PCR-SSP typing association studies particularly where NA2 was associated with disease outcome. The risk associated allotype NA2*03 found in the Ghanaian population, differs from the classical NA2 by an asparagine (N) to aspartic acid (D) substitution at position 82 of the FcγRIIIB protein. This N82D substitution results in a loss of one potential N-glycosylation site of NA2. It is conceivable, that the NA2*03 variant would have one-less (ie. 5) potential N-glycosylation sites. NA2 shows a reduced capacity to facilitate phagocytosis, respiratory burst and degranulation responses compared to NA1, a property attributed to the extensive glycosylation in NA2 than in NA1 [9], [41]. Thus, it would be expected that the NA2*03 variant possessing one-less glycosylation site compared to NA2 should have a higher affinity for IgG1 and IgG3 than NA2 and hence be the more efficient receptor. However, no study has as yet investigated the binding affinity of the NA2*03 variant for IgG1 and IgG3 or the possible conformational changes due to the replacement of a potential glycosylated group (p.82N) with an unglycosylated charged group (p.82D). N-glycosylation is a common feature of many membrane-bound and extracellular proteins in animals and the carbohydrate groups are considered crucial for biological functions such as proper protein folding [42], protein stability and solubility [43], ligand binding affinity [44], signal transduction [45], and immunogenicity [46]. Thus, it is plausible that the p.N82D substitution in the NA2*03 variant may alter an important biological mechanism critical for neutrophil respiratory burst and hence predispose to clinical malaria. Further studies are needed to delineate the possible functional consequences of the NA2*03 polymorphism in FcγRIIIB.

The Tajima's D analysis showed exon 3 of *FCGR3B* to be under selection pressure in both the Ghanaian and the Danish populations; however, it is not clear what may be driving the selection in these different populations. Malaria has been considered the strongest known selective pressure in the recent history of the human genome [47]. There was a higher frequency of the c.233A allele in malaria endemic populations and high pairwise F_ST_ indices (0.098≤F_ST_≤0.194) between the malaria versus non-malaria endemic populations. These observations support the notion that malaria may be a significant contributor to the selection pressure, at least in the Ghanaian and African (AFR) populations. However, other possible factors that could explain the selection in both the malaria and non-malaria endemic populations may include inflammatory diseases such as rheumatoid arthritis [48] or periodontal diseases (PD). PD is a widespread condition and several studies have associated FcγRIIIB polymorphisms with PD in different populations [49]. In animal studies, PD has been shown to contribute to perinatal mortality [50]. A recent study in humans concluded that, in cases of extreme prematurity, maternal PD may be a significant contributor to perinatal mortality [51]. Given the contribution of PD in perinatal mortality and the association of FcγRIIIB polymorphisms in the pathogenesis of PD, we speculate that PD may contribute to the selection pressure acting on exon 3 of *FCGR3B*. It is however, worth noting that the present data does not clearly show which modes of natural selection may be at play in these two populations. Further studies are needed to clearly define the forces of selection on *FCGR3B*-exon 3 in these populations.

In conclusion, the present study has identified the c.233A allele of *FCGR3B*-c.233C>A (rs5030738) and the FcγRIIIB-SH (CTGAAA) haplotype to be associated with protection from clinical malaria. The FcγRIIIB-NA2*03 (CTGCGA) variant of FcγRIIIB-NA2 (CTGCAA) was associated with susceptibility to clinical malaria in the Ghanaian population. The study provides the justification for a more detailed functional characterisation of the FcγRIIIB-SH and FcγRIIIB-NA2*03 haplotypes in relation to neutrophil functionality especially in respiratory burst activity.

## Materials and Methods

### Ethics Statement

Ethical approval for the study was given by the Institutional Review Board of the Noguchi Memorial Institute for Medical Research (NMIMR) of the University of Ghana, Accra, Ghana. Written informed consent was given by the parents and guardians of children before they were enrolled into the study. Ethical approval for Danish blood donor samples was given by the Scientific Ethics Committee of Copenhagen and Frederiksberg, Denmark. DNA samples from a total of 132 anonymous Danish blood, obtained for control purposes from Copenhagen University Hospital, were analysed in order to allow population comparisons of genotype distributions. These individuals are resident of central Copenhagen and provided written consent to have a small portion of their blood stored, anonymously, and used for research purposes. Blood donors in Denmark must be between the ages of 18 and 60. All data were analysed anonymously.

### Study area, population and baseline sampling

The study was conducted in Asutsuare (ASU) (about 120 km north-east of Accra) and five neighbouring villages: Kewum-Atrobinya (KEW), Avakpo (AVA), Mafikorpe (MAF), Osuwem (OSU) and Volivo (VOL) of the Damgbe West District of the Greater-Accra Region of Ghana. The villages are only about 2-5 km apart. Like many other parts of Ghana, the climate of the area is characterised by two major seasons: a dry or the harmattan season (December to March) and a wet or the rainy season (June to August), however, there are also some few rains in November and early December, just before the onset of the harmattan season. Malaria transmission occurs throughout the year but peaks during and after the rains (September and January). *P. falciparum* constitutes 98% of all infection with the remaining 2% due to *P. malariae* and *P. ovale* [52]. The population is predominantly of the Ga-Adangbe ethnic origin but is interspersed with other ethnic groups such as the Ewes and the Akans. There are two health centres serving all these communities: Osudoku Community Health Centre at Asutsuare and the Osuwem Community Health Centre. In addition, the Akuse Hospital, about 10 km away serves as a referral hospital for cases beyond the capacity of the community health centres.

Altogether, 798 children (aged 1 to 12 years old) were enrolled and were followed up actively and passively for malaria case detection in a 42 week longitudinal cohort study. Genomic DNA for analysis was available for 585 of the 669 children who successfully completed the follow up without missing at most three successive weekly visits. Of these 585 children, there were 316 singletons, 91 families with two children enrolled, 17 families with 3 children enrolled and 9 families with 4 children enrolled.. At baseline (enrolment), 5 ml EDTA-anticoagulated venous blood and thick and thin film blood slides were obtained from all individuals prior to the malaria transmission season (May 2008) for baseline immunological and parasitological determinations. Blood group and sickle cell status of each individual were determined by a commercial blood grouping kit (Biotec Laboratories Limited, UK) and the sodium metabisulphite test respectively and haemoglobin (Hb) level was measured using the Hemocue-Hb 201 (Angelhom, Sweden). Blood was centrifuged to separate plasma and peripheral blood mononuclear cells (PBMCs) and stored at −80°C and in liquid nitrogen respectively. The thick and thin blood film slides were stained with Giemsa and examined for baseline asymptomatic parasitaemia. A slide was negative if no parasite was visualised in 200 oil fields of a thick film. For slides that were positive, parasites were counted per 200 white blood cells (WBCs) and parasite densities calculated by assuming 8,000 WBCs/µl blood. A standardised questionnaire was used to obtain epidemiological, anthropometrical and clinical data of all study participants during the enrolment.

### Danish Donors

DNA samples from a total of 132 anonymous Danish blood donors, obtained for control purposes, were analysed in order to allow population comparisons of genotype distributions. These individuals are resident of central Copenhagen and provided consent to have a small portion of their blood stored, anonymously, and used for research purposes. Blood donors in Denmark must be between the ages of 18 and 60 [33].

### Parasitological and Clinical Surveillance

Parasitological surveillance for malaria infection was carried out monthly for each study participant during the follow-up period. This involved obtaining thick and thin blood film slides from finger pricks by trained medical personnel. In addition, about 500 µl of blood was collected during the monthly finger pricking to obtain plasma for immunological analyses. The remaining packed cells were stored at −80°C for DNA purification for genetic analyses. The active case detection surveillance comprised weekly visits to each participant's home, where a morbidity questionnaire (investigating symptoms occurring in the preceding week) was administered by trained Field Assistants. The presence or absence of fever (measured axillary temperature of ≥37.5°C, or reported) was ascertained. Study participants complaining of symptoms suggestive of malaria were referred for treatment at the respective health centres. In the passive case detection surveillance, visits by participants to the health centres without prior referral from a field assistant were documented. In both the active and passive case detection, Hb level was measured and thick and thin blood films were obtained from study participants with febrile temperature (>37.5°C) or reported febrile temperature prior to treatment. Clinical malaria was defined as slide positive for any asexual *P. falciparum* parasitaemia with at least one other sign of malaria such as vomiting, diarrhoea, or malaise. Malaria was treated with artesunate-amodiaquine combined dose therapy which was the recommended standard treatment for malaria in Ghana. At the end of the study, on the basis of the clinical and parasitological data obtained, the study population was divided into three groups: (1) those susceptible, in which parasitaemia was associated with febrile disease, and (2) those apparently protected against clinical manifestation despite parasitaemia and (3) those apparently protected against clinical manifestation without detectable parasitaemia by microscopy.

### *FCGR2A genotyping*

Genomic DNA was purified from packed cell samples using the Maxwell®16 system (Promega, Madison, USA) following manufacturer's guidelines. Genotyping of FcγRIIA-p.H166R was done by the gene specific polymerase chain reaction (PCR) amplification followed by allele specific restriction enzyme digestion (ASRED) method [53]. The final B*st*UI restriction digestion products were visualized as 343 bp (H allele) and 322 bp (R allele) bands on 3% agarose (SeaKem®GTG® Agarose, ME) with ethidium bromide (AppliChem, Damstadt, Germany) staining. Both fragments were present for heterozygous individuals.

### FCGR3B sequencing

We designed a protocol to specifically amplify and sequence exon 3 of the *FCGR3B* gene from genomic DNA. First, an approximately 4.3-kb fragment of the *FCGR3B* gene using the sense primer (5′-CTCCATTGCGAGACTTCAGAT-3′) placed in exon 1 and the antisense primer (5′-CGTGGTTTCTAAGGTGTCACAGG-3′), positioned within intron 3. A 30-cycle amplification process consisting of denaturation at 95°C for 30 s, annealing at 63°C for 30 s and extension at 72°C for 5 mins was performed using *PfuUltra*® high-fidelity DNA polymerase (Stratagene, USA). The product was gel purified using E.Z.N.A® Gel Extraction Kit (Omega Bio-Tek, Inc., GA) and used as template in a nested PCR to amplify exon 3 of *FCGR3B* with the M13 tagged (in lower case) sense (5′-tgtaaaacgacggccagtGTCAGCTTCATGGTCTTGGATTG-3′) and antisense (5′-caggaaacagctatgaccACACATTCACATTGTATGCACTCCA-3′) primers. The 38 cycle amplification consisted of denaturation at 94°C for 30 s, annealing at 58°C for 30 s and extension at 72°C for 45 sec using TEMPase hotstart DNA polymerase (Biomol, Germany). The nested PCR product was then sequenced with M13 primers. The low affinity *FCGR* locus contains regions of copy number variation (CNV) which can alter receptor expression and leukocyte responses to IgG. However, previous *FCGR3B* CNV data determined by the SALSA® multiplex ligation probe amplification (MLPA®) kit P110-B1/P111-B1 FCGR (Lot 0210, 0409; v.08) (MRC Holland) on the same samples in the present study found no association with and clinical malaria (Adu *et al.*, unpublished).

### Statistical analysis

Demographic and clinical characteristics of the study population were compared across village of residence (χ^2^-test). Since the risk of clinical malaria is known to be high in children aged 5 years and below, age was modelled as a categorical variable with two levels (1–5 years, and 6–12 years). Logistic regression analyses and likelihood ratio tests were carried out to evaluate the association of the variables, age group, sex, sickle cell status, blood group, use of bed net and ethnic group with clinical malaria. The genotype distribution of the SNPs was compared across ethnic groups (χ^2^-test). To test Hardy Weinberg equilibrium (HWE), we used the data of n = 267 children who had diploid copies of *FCGR3B*, were the first sibling in a family and were not susceptible to clinical malaria in the observation period. The exact test (HWE.exact) implemented in the R-package ‘genetics v.1.3.6’ (http://CRAN.R project.org/package = genetics) was used. For each of the SNPs, the minor allele (single marker) association with clinical malaria was calculated under the three genetic models of inheritance (additive, recessive and dominant) using logistic regression adjusting for age group, sex, sickle cell status, blood group, use of bed net and ethnic group. Two different control groups were defined: (1) all individuals with no malaria episodes but with definitive exposure as indicated by parasites detectable by microscopy during the follow up, plus all individuals with no malaria episodes but without parasites detectable by microscopy and (2) only individuals with no malaria episodes but with definitive exposure as indicated by parasites detectable by microscopy during the follow up. The second analyses involving control group (2) was to account for any possible confounding due to heterogeneity in exposure. This was because, the group comprising individuals apparently protected but without detectable parasitaemia by microscopy may also include individuals who did not have malaria due to lack of exposure to the parasite during the follow up period. Additional sensitivity SNP association analyses (logistic regression) were performed with a generalized estimating equation (GEE) approach to correct all confidence limits and *p*-values for the family structure in the data. Linkage disequilibrium (LD) in the *FCGR3B* SNPs and visualisation of pairwise r^2^ LD values were evaluated using Haploview v. 4.2 [54]. Association of *FCGR3B* haplotypes with clinical malaria was analysed using the Hapassoc package v. 1.2–4 in R [55]. The pooling tolerance was set to 0.03 in order to restrict the association analysis to haplotypes whose frequency exceeded 3% in the population. The haplotype with the highest frequency was used as the reference. Comparison to the other haplotypes was performed using nested logistic regression adjusting for age group, gender, sickle cell status, blood group, use of bed net and ethnic group. Except for the LD and pairwise r^2^ visualization, all statistical analyses were performed using R v. 2.13.2 (http://www.R-project.org). DnaSP v. 5.10 (http://www.ub.edu/dnasp/) was used to estimate Tajima's D for exon 3 of *FCGR3B* the Ghanaian and Danish populations. The window length for the Tajima's sliding window analysis was 50 with step size of 10 nucleotides. Genotyping data for rs5030738 was retrieved from the 1000 Genomes Project database (http://browser.1000genomes.org/Homo_sapiens/Variation/Population?db=coreg=ENSG00000162747r=1:161592986-161601753t=ENST00000531221v=rs5030738vdb=variationvf=8673417); for the African (AFR); Ad Mixed American (AMR) and European (EUR) super populations and allele frequencies compared among all 5 populations. Pairwise F_ST_ distances were calculated for all 5 populations using the POPTREE2 software [56].

## Supporting Information

Table S1 **Distribution of** ***FCGR2A*** **and** ***FCGR3B*** **genotypes among the ethnic groups in the study population.** * Comprises individuals from Akan, Hausa and Fulani ethnic groups
(DOC)

Table S2 **Univariate analyses of** ***FCGR2A*** **and** ***FCGR3B*** **genotypes association with clinical malaria.** ^a^ encodes the FcγRIIA-166H/R polymorphism. ^b^ adjusted for age groups, sex, sickle cell status, blood group, bed net use and ethnic group
(DOC)

Table S3 **Single marker association of** ***FCGR3B*** **alleles with clinical malaria using sub-set of controls.** Odds ratio (OR) and 95% confidence intervals (CI) were determined using multivariate logistic regression controlling for age, gender, ethnicity, sickle-cell status, *FCGR3B* copy number, blood group, family structure and use of bed net. MAF: minor allele frequency. **^¥^**All individuals who never had malaria despite parasitaemia at any time point during the study
(DOC)

## References

[1] World Health Organisation (2010) World Malaria Report 2010, Geneva, Switzerland. 1–238 p.

[2] Dodoo D, Theisen M, Kurtzhals JA, Akanmori BD, Koram KA, et al (2000) Naturally acquired antibodies to the glutamate-rich protein are associated with protection against Plasmodium falciparum malaria. J InfectDis 181: 1202–1205. 10.1086/315341 10720556

[3] Oeuvray C, Theisen M, Rogier C, Trape JF, Jepsen S, et al (2000) Cytophilic immunoglobulin responses to Plasmodium falciparum glutamate-rich protein are correlated with protection against clinical malaria in Dielmo, Senegal. InfectImmun 68: 2617–2620. 10.1128/iai.68.5.2617-2620.2000 PMC97467 10768952

[4] Soe S, Theisen M, Roussilhon C, Aye KS, Druilhe P (2004) Association between protection against clinical malaria and antibodies to merozoite surface antigens in an area of hyperendemicity in Myanmar: complementarity between responses to merozoite surface protein 3 and the 220-kilodalton glutamate-rich protein. InfectImmun 72: 247–252. 10.1128/IAI.72.1.247-252.2004 PMC343946 14688102

[5] Bouharoun-Tayoun H, Oeuvray C, Lunel F, Druilhe P (1995) Mechanisms underlying the monocyte-mediated antibody-dependent killing of Plasmodium falciparum asexual blood stages. JExpMed 182: 409–418. 10.1084/jem.182.2.409 PMC2192140 7629503

[6] Shi YP, Udhayakumar V, Oloo AJ, Nahlen BL, Lal AA (1999) Differential effect and interaction of monocytes, hyperimmune sera, and immunoglobulin G on the growth of asexual stage Plasmodium falciparum parasites. Am J Trop Med Hyg 60: 135–141. 9988337 10.4269/ajtmh.1999.60.135

[7] Joos C, Marrama L, Polson HE, Corre S, Diatta AM, et al (2010) Clinical protection from falciparum malaria correlates with neutrophil respiratory bursts induced by merozoites opsonized with human serum antibodies. PLoSOne 5: e9871. 10.1371/journal.pone.0009871 PMC2845614 20360847

[8] Perussia B, Dayton ET, Lazarus R, Fanning V, Trinchieri G (1983) Immune interferon induces the receptor for monomeric IgG1 on human monocytic and myeloid cells. JExpMed 158: 1092–1113. 10.1084/jem.158.4.1092 PMC2187379 6225822

[9] Salmon JE, Edberg JC, Brogle NL, Kimberly RP (1992) Allelic polymorphisms of human Fc gamma receptor IIA and Fc gamma receptor IIIB. Independent mechanisms for differences in human phagocyte function. J Clin Invest 89: 1274–1281. 1532589 10.1172/JCI115712 PMC442988

[10] Ravetch JV (2003) In Fundamental Immunology (ed. Paul, W. E.). Lippincott-Raven 685–700.

[11] Ravetch JV, Bolland S (2001) IgG Fc receptors. AnnuRevImmunol 19: 275–290. 10.1146/annurev.immunol.19.1.275 11244038

[12] Boross P, van de Poel K, Van de Winkel JGJ, Leusen JHW (2008) Fc Receptors. Encyclopedia of Life Sciences (ELS). Chichester: John Wiley & Sons, Ltd 1–8.

[13] Mitchell MA, Huang MM, Chien P, Indik ZK, Pan XQ, et al (1994) Substitutions and deletions in the cytoplasmic domain of the phagocytic receptor Fc gamma RIIA: effect on receptor tyrosine phosphorylation and phagocytosis. Blood 84: 1753–1759. 7521687

[14] Salmon JE, Millard SS, Brogle NL, Kimberly RP (1995) Fc gamma receptor IIIb enhances Fc gamma receptor IIa function in an oxidant-dependent and allele-sensitive manner. JClinInvest 95: 2877–2885. 10.1172/JCI117994 PMC295975 7769129

[15] Clark IA, Hunt NH (1983) Evidence for reactive oxygen intermediates causing hemolysis and parasite death in malaria. Infect Immun 39: 1–6. 6822409 10.1128/iai.39.1.1-6.1983 PMC347899

[16] Allison AC, Eugui EM (1983) The role of cell-mediated immune responses in resistance to malaria, with special reference to oxidant stress. Annu Rev Immunol 1: 361–392. 6100538 10.1146/annurev.iy.01.040183.002045

[17] Greve B, Lehman LG, Lell B, Luckner D, Schmidt-Ott R, et al (1999) High oxygen radical production is associated with fast parasite clearance in children with Plasmodium falciparum malaria. J Infect Dis 179: 1584–1586. 10228089 10.1086/314780

[18] Warmerdam PA, van de Winkel JG, Gosselin EJ, Capel PJ (1990) Molecular basis for a polymorphism of human Fc gamma receptor II (CD32). J Exp Med 172: 19–25. 2141627 10.1084/jem.172.1.19 PMC2188138

[19] Parren PW, Warmerdam PA, Boeije LC, Arts J, Westerdaal NA, et al (1992) On the interaction of IgG subclasses with the low affinity Fc gamma RIIa (CD32) on human monocytes, neutrophils, and platelets. Analysis of a functional polymorphism to human IgG2. J Clin Invest 90: 1537–1546. 1401085 10.1172/JCI116022 PMC443201

[20] Schuldt K, Esser C, Evans J, May J, Timmann C, et al (2010) FCGR2A functional genetic variant associated with susceptibility to severe malarial anaemia in Ghanaian children. J Med Genet 47: 471–475. 19965803 10.1136/jmg.2009.073643

[21] Bux J, Kissel K, Hofmann C, Santoso S (1999) The use of allele-specific recombinant Fc gamma receptor IIIb antigens for the detection of granulocyte antibodies. Blood 93: 357–362. 9864181

[22] Najera JA (1989) Malaria and the work of WHO. BullWorld Health Organ 67: 229–243. PMC2491250 2670294

[23] Ory PA, Clark MR, Kwoh EE, Clarkson SB, Goldstein IM (1989) Sequences of complementary DNAs that encode the NA1 and NA2 forms of Fc receptor III on human neutrophils. J Clin Invest 84: 1688–1691. 2478590 10.1172/JCI114350 PMC304039

[24] Kimberly RP, Salmon JE, Edberg JC (1995) Receptors for immunoglobulin G. Molecular diversity and implications for disease. Arthritis Rheum 38: 306–314. 7880183 10.1002/art.1780380303

[25] Bux J, Stein EL, Bierling P, Fromont P, Clay M, et al (1997) Characterization of a new alloantigen (SH) on the human neutrophil Fc gamma receptor IIIb. Blood 89: 1027–1034. 9028335

[26] Koene HR, Kleijer M, Roos D, de Haas M, dem Borne AE (1998) Fc gamma RIIIB gene duplication: evidence for presence and expression of three distinct Fc gamma RIIIB genes in NA(1+,2+)SH(+) individuals. Blood 91: 673–679. 9427724

[27] de Haas M, Kleijer M, van Zwieten R, Roos D, von dem Borne AE (1995) Neutrophil Fc gamma RIIIb deficiency, nature, and clinical consequences: a study of 21 individuals from 14 families. Blood 86: 2403–2413. 7662988

[28] Matsuo K, Procter J, Stroncek D (2000) Variations in genes encoding neutrophil antigens NA1 and NA2. Transfusion 40: 645–653. 10864983 10.1046/j.1537-2995.2000.40060645.x

[29] Tong Y, Jin J, Yan L, Neppert J, Marget M, et al (2003) FCGR3B gene frequencies and FCGR3 variants in a Chinese population from Zhejiang Province. Ann Hematol 82: 574–578. 12898191 10.1007/s00277-003-0725-y

[30] Terzian CC, Chiba AK, Santos VC, Silva NP, Bordin JO (2011) FCGR3B*03 allele inheritance pattern in Brazilian families and some new variants of gene FCGR3B. Transfusion 10.1111/j.1537-2995.2011.03326.x 21895673

[31] Omi K, Ohashi J, Patarapotikul J, Hananantachai H, Naka I, et al (2002) Fcgamma receptor IIA and IIIB polymorphisms are associated with susceptibility to cerebral malaria. Parasitol Int 51: 361–366. 12421634 10.1016/s1383-5769(02)00040-5

[32] Ouma C, Davenport GC, Garcia S, Kempaiah P, Chaudhary A, et al (2011) Functional haplotypes of Fc gamma (Fcgamma) receptor (FcgammaRIIA and FcgammaRIIIB) predict risk to repeated episodes of severe malarial anemia and mortality in Kenyan children. Hum Genet 10.1007/s00439-011-1076-8 PMC3258363 21818580

[33] Adu B, Dodoo D, Adukpo S, Gyan BA, Hedley PL, et al (2011) Polymorphisms in the RNASE3 gene are associated with susceptibility to cerebral malaria in Ghanaian children. PLoS One 6: e29465. 22216286 10.1371/journal.pone.0029465 PMC3246477

[34] Schuldt K, Kretz CC, Timmann C, Sievertsen J, Ehmen C, et al (2011) A -436C>A polymorphism in the human FAS gene promoter associated with severe childhood malaria. PLoS Genet 7: e1002066. 21625619 10.1371/journal.pgen.1002066 PMC3098189

[35] Bejon P, Warimwe G, Mackintosh CL, Mackinnon MJ, Kinyanjui SM, et al (2009) Analysis of immunity to febrile malaria in children that distinguishes immunity from lack of exposure. Infect Immun 77: 1917–1923. 19223480 10.1128/IAI.01358-08 PMC2681775

[36] Theisen M, Soe S, Oeuvray C, Thomas AW, Vuust J, et al (1998) The glutamate-rich protein (GLURP) of Plasmodium falciparum is a target for antibody-dependent monocyte-mediated inhibition of parasite growth in vitro. InfectImmun 66: 11–17. 10.1128/iai.66.1.11-17.1998 PMC107852 9423833

[37] Vinten-Johansen J (2004) Involvement of neutrophils in the pathogenesis of lethal myocardial reperfusion injury. Cardiovasc Res 61: 481–497. 14962479 10.1016/j.cardiores.2003.10.011

[38] Lettre G, Lange C, Hirschhorn JN (2007) Genetic model testing and statistical power in population-based association studies of quantitative traits. Genet Epidemiol 31: 358–362. 17352422 10.1002/gepi.20217

[39] Bux J, Stein EL, Santoso S, Mueller-Eckhardt C (1995) NA gene frequencies in the German population, determined by polymerase chain reaction with sequence-specific primers. Transfusion 35: 54–57. 7998071 10.1046/j.1537-2995.1995.35195090663.x

[40] Blum KS, Tong Y, Siebert R, Marget M, Humpe A, et al (2009) Evidence for gene recombination in FCGR3 gene variants. Vox Sang 97: 69–76. 19320901 10.1111/j.1423-0410.2009.01178.x

[41] Salmon JE, Edberg JC, Kimberly RP (1990) Fc gamma receptor III on human neutrophils. Allelic variants have functionally distinct capacities. JClinInvest 85: 1287–1295. 10.1172/JCI114566 PMC296565 1690757

[42] Tifft CJ, Proia RL, Camerini-Otero RD (1992) The folding and cell surface expression of CD4 requires glycosylation. J Biol Chem 267: 3268–3273. 1737783

[43] Niu L, Heaney ML, Vera JC, Golde DW (2000) High-affinity binding to the GM-CSF receptor requires intact N-glycosylation sites in the extracellular domain of the beta subunit. Blood 95: 3357–3362. 10828016

[44] Szecowka J, Tai LR, Goodman HM (1990) Effects of tunicamycin on growth hormone binding in rat adipocytes. Endocrinology 126: 1834–1841. 2318146 10.1210/endo-126-4-1834

[45] Leconte I, Auzan C, Debant A, Rossi B, Clauser E (1992) N-linked oligosaccharide chains of the insulin receptor beta subunit are essential for transmembrane signaling. J Biol Chem 267: 17415–17423. 1324936

[46] Feizi T, Childs RA (1987) Carbohydrates as antigenic determinants of glycoproteins. Biochem J 245: 1–11. 2444214 10.1042/bj2450001 PMC1148075

[47] Kwiatkowski DP (2005) How malaria has affected the human genome and what human genetics can teach us about malaria. Am J Hum Genet 77: 171–192. 16001361 10.1086/432519 PMC1224522

[48] Morgan AW, Barrett JH, Griffiths B, Subramanian D, Robinson JI, et al (2006) Analysis of Fcgamma receptor haplotypes in rheumatoid arthritis: FCGR3A remains a major susceptibility gene at this locus, with an additional contribution from FCGR3B. Arthritis Res Ther 8: R5. 16356189 10.1186/ar1847 PMC1526569

[49] Dimou NL, Nikolopoulos GK, Hamodrakas SJ, Bagos PG (2010) Fcgamma receptor polymorphisms and their association with periodontal disease: a meta-analysis. J Clin Periodontol 37: 255–265. 20149216 10.1111/j.1600-051X.2009.01530.x

[50] Han YW, Redline RW, Li M, Yin L, Hill GB, et al (2004) Fusobacterium nucleatum induces premature and term stillbirths in pregnant mice: implication of oral bacteria in preterm birth. Infect Immun 72: 2272–2279. 15039352 10.1128/IAI.72.4.2272-2279.2004 PMC375172

[51] Shub A, Wong C, Jennings B, Swain JR, Newnham JP (2009) Maternal periodontal disease and perinatal mortality. Aust N Z J Obstet Gynaecol 49: 130–136. 19441161 10.1111/j.1479-828x.2009.00953.x

[52] Ministry of Health (1992) Malaria Action Plan 1993–1997. Accra: Epidemiology Division Ministry of Health, Ghana, Accra.

[53] Jiang XM, Arepally G, Poncz M, McKenzie SE (1996) Rapid detection of the Fc gamma RIIA-H/R 131 ligand-binding polymorphism using an allele-specific restriction enzyme digestion (ASRED). J Immunol Methods 199: 55–59. 8960098 10.1016/s0022-1759(96)00164-0

[54] Barrett JC, Fry B, Maller J, Daly MJ (2005) Haploview: analysis and visualization of LD and haplotype maps. Bioinformatics 21: 263–265. 15297300 10.1093/bioinformatics/bth457

[55] Burkett K, Graham J, McNeney B (2006) hapassoc: Software for likelihood inference of trait associations with SNP haplotypes and other attributes. J Stat Soft 16: 1–19. 10.1159/000081447 15583426

[56] Takezaki N, Nei M, Tamura K (2010) POPTREE2: Software for constructing population trees from allele frequency data and computing other population statistics with Windows interface. Mol Biol Evol 27: 747–752. 20022889 10.1093/molbev/msp312 PMC2877541

