# Supplementary material for: Fc Gamma Receptor IIIB (FcγRIIIB) Polymorphisms Are Associated with Clinical Malaria in Ghanaian Children
Source: PLoS One. 2012 Sep 25;7(9):e46197. doi: 10.1371/journal.pone.0046197 (PMC3458101; doi:10.1371/journal.pone.0046197)
Supplement: Table S1 — Distribution of FCGR2A and FCGR3B genotypes among the ethnic groups in the study population. * Comprises individuals from Akan, Hausa and Fulani ethnic groups (DOC) [file pone.0046197.s001.doc]

**Table S1.** Distribution of *FCGR2A* and *FCGR3B* genotypes among the ethnic groups in the study population

|  | **Ga-Adangbe (n=430)** | **Ewe (n=76)** | **Other* (n=79)** | ***p*-value** |
| --- | --- | --- | --- | --- |
| ***FCGR2A*** |  |  |  |  |
| **c.497A>G** |  |  |  |  |
| AA | 67 | 15 | 12 |  |
| AG | 203 | 42 | 49 |  |
| GG | 160 | 19 | 18 | 0.038 |
| ***FCGR3B*** |  |  |  |  |
| **c.108C>G** |  |  |  |  |
| CC | 126 | 24 | 33 |  |
| CG | 175 | 31 | 19 |  |
| GG | 129 | 21 | 27 | 0.065 |
| **c.114T>C** |  |  |  |  |
| CC | 112 | 20 | 25 |  |
| CT | 187 | 31 | 20 |  |
| TT | 131 | 25 | 34 | 0.051 |
| **c.194A>G** |  |  |  |  |
| AA | 81 | 24 | 19 |  |
| AG | 173 | 24 | 20 |  |
| GG | 176 | 28 | 40 | 0.017 |
| **c.233C>A** |  |  |  |  |
| AA | 38 | 5 | 7 |  |
| AC | 116 | 14 | 17 |  |
| CC | 276 | 57 | 55 | 0.40 |
| **c.244A>G** |  |  |  |  |
| AA | 124 | 26 | 24 |  |
| AG | 168 | 28 | 26 |  |
| GG | 138 | 22 | 29 | 0.72 |
| **c.316A>G** |  |  |  |  |
| AA | 250 | 40 | 53 |  |
| AG | 132 | 24 | 13 |  |
| GG | 48 | 12 | 13 | 0.071 |

***** Comprises individuals from Akan, Hausa and Fulani ethnic groups
