# Supplementary material for: Fc Gamma Receptor IIIB (FcγRIIIB) Polymorphisms Are Associated with Clinical Malaria in Ghanaian Children
Source: PLoS One. 2012 Sep 25;7(9):e46197. doi: 10.1371/journal.pone.0046197 (PMC3458101; doi:10.1371/journal.pone.0046197)
Supplement: Table S2 — Univariate analyses of FCGR2A and FCGR3B genotypes association with clinical malaria. a encodes the FcγRIIA-166H/R polymorphism. b adjusted for age groups, sex, sickle cell status, blood group, bed net use and ethnic group (DOC) [file pone.0046197.s002.doc]

**Table S2. Univariate analyses of *FCGR2A* and *FCGR3B* genotypes association with clinical malaria**

|  | **Protected** | **Susceptible** | **OR(95%CI)^b^** | ***p*-value^b^** | **LR test (*p*-value)** |
| --- | --- | --- | --- | --- | --- |
| ***FCGR2A*** |  |  |  |  |  |
| **c.497A>G** ^a^ |  |  |  |  |  |
| AA | 87 | 7 | 1 |  |  |
| AG | 266 | 28 | 1.27 (0.55-3.28) | 0.60 |  |
| GG | 180 | 17 | 1.06 (0.43-2.88) | 0.90 | 0.80 |
| ***FCGR3B*** |  |  |  |  |  |
| **c.108C>G** |  |  |  |  |  |
| CC | 164 | 19 | 1 |  |  |
| CG | 211 | 14 | 0.57 (0.27-1.17) | 0.13 |  |
| GG | 158 | 19 | 1.04 (0.52-2.08) | 0.91 | 0.18 |
| **c.114T>C** |  |  |  |  |  |
| CC | 142 | 15 | 1 |  |  |
| CT | 220 | 18 | 0.76 (0.37-1.61) | 0.48 |  |
| TT | 171 | 19 | 1.04 (0.50-2.18) | 0.92 | 0.65 |
| **c.194A>G** |  |  |  |  |  |
| AA | 112 | 12 | 1 |  |  |
| AG | 202 | 15 | 0.63 (0.28-1.45) | 0.27 |  |
| GG | 219 | 25 | 0.95 (0.46-2.06) | 0.89 | 0.41 |
| **c.233C>A** |  |  |  |  |  |
| AA | 49 | 1 | 1 |  |  |
| AC | 139 | 8 | 2.86 (0.50-54.09) | 0.33 |  |
| CC | 345 | 43 | 6.49 (1.36-116.99) | 0.068 | 0.0088 |
| **c.244A>G** |  |  |  |  |  |
| AA | 164 | 10 | 1 |  |  |
| AG | 200 | 22 | 1.77 (0.83-4.06) | 0.15 |  |
| GG | 169 | 20 | 1.93 (0.89-4.48) | 0.11 | 0.21 |
| **c.316A>G** |  |  |  |  |  |
| AA | 308 | 35 | 1 |  |  |
| AG | 158 | 11 | 0.63 (0.29-1.25) | 0.20 |  |
| GG | 67 | 6 | 0.84 (0.31-1.96) | 0.71 | 0.42 |

^a^ encodes the FcγRIIA-166H/R polymorphism. ^b^ adjusted for age groups, sex, sickle cell status, blood group, bed net use and ethnic group
