# Supplementary material for: Fc Gamma Receptor IIIB (FcγRIIIB) Polymorphisms Are Associated with Clinical Malaria in Ghanaian Children
Source: PLoS One. 2012 Sep 25;7(9):e46197. doi: 10.1371/journal.pone.0046197 (PMC3458101; doi:10.1371/journal.pone.0046197)
Supplement: Table S3 — Single marker association of FCGR3B alleles with clinical malaria using sub-set of controls. Odds ratio (OR) and 95% confidence intervals (CI) were determined using multivariate logistic regression controlling for age, gender, ethnicity, sickle-cell status, FCGR3B copy number, blood group, family structure and use of bed net. MAF: minor allele frequency. ¥All individuals who never had malaria despite parasitaemia at any time point during the study (DOC) [file pone.0046197.s003.doc]

**Table S3.** Single marker association of *FCGR3B* alleles with clinical malaria using sub-set of controls

|  |  | **MAF** | | **Additive model** | | **Recessive model** | | **Dominant model** | |
| --- | --- | --- | --- | --- | --- | --- | --- | --- | --- |
| **SNP ID** | **Minor Allele** | **Protected^¥^** | **Susceptible** | **OR (95% CI)** | ***p*-value** | **OR (95% CI)** | ***p*-value** | **OR (95% CI)** | ***p*-value** |
| rs403016 | C | 0.47 | 0.50 | 0.98 (0.57-1.70) | 0.95 | 1.40 (0.56-3.55) | 0.46 | 0.69 (0.28-1.70) | 0.42 |
| rs447536 | C | 0.50 | 0.46 | 0.92 (0.52-1.63) | 0.77 | 1.31 (0.50-3.43) | 0.58 | 0.61 (0.24-1.52) | 0.29 |
| rs448740 | A | 0.39 | 0.38 | 1.01 (0.58-1.74) | 0.98 | 1.54 (0.58-4.06) | 0.39 | 0.74 (0.30-1.84) | 0.52 |
| rs5030738 | A | 0.25 | 0.10 | 0.32 (0.14-0.72) | 0.0060 | 0.18 (0.02-1.66) | 0.13 | 0.25 (0.10-0.66) | 0.0051 |
| rs428888 | A | 0.52 | 0.40 | 0.62 (0.34-1.13) | 0.12 | 0.58 (0.20-1.67) | 0.31 | 0.45 (0.18-1.14) | 0.092 |
| rs2290834 | G | 0.25 | 0.22 | 0.90 (0.47-1.68) | 0.73 | 1.51 (0.40-5.65) | 0.55 | 0.68 (0.28-1.65) | 0.39 |

Odds ratio (OR) and 95% confidence intervals (CI) were determined using multivariate logistic regression controlling for age, gender, ethnicity, sickle-cell status, *FCGR3B* copy number, blood group, family structure and use of bed net. MAF: minor allele frequency. **^¥^**All individuals who never had malaria despite parasitaemia at any time point during the study
